# Application of Spruce Bark Biochar Minimizes Nitrogen and Carbon Leaching from an Eastern Newfoundland Podzolic Soil

**DOI:** 10.3390/plants14233687

**Published:** 2025-12-03

**Authors:** Riad O. Eissa, Lordwin Jeyakumar, David B. McKenzie, Jianghua Wu

**Affiliations:** 1 Faculty of Agriculture, Department of Soil and Water, Sebha University, Sebha P.O. Box 18758, Libya; riad.eissa@sebhau.edu.ly; 2 Nova Scotia Environment and Climate Change, 1903 Barrington (2nd Floor), Suite 2085, Halifax, NS B3J 2P8, Canada; lordwin.jeyakumar@novascotia.ca; 3 Agriculture and Agri-Food Canada, St. John’s Research and Development Centre, St. John’s, NL A1E 6J5, Canada; david.mckenzie@agr.gc.ca; 4 Environmental Science, Memorial University of Newfoundland, St. John’s, NL A1C 5S7, Canada; 5 Environment and Sustainability, School of Science and the Environment, Grenfell Campus, Memorial University of Newfoundland, Corner Brook, NL A2H 5G4, Canada; 6 Key Laboratory of Ecosystem Carbon Sources and Sinks, China Meteorological Administration (ECSS-CMA), School of Ecology and Applied Meteorology, Nanjing University of Information Science & Technology, Nanjing 210044, China

**Keywords:** agriculture, ammonium, biochar, dissolved organic carbon, nitrate

## Abstract

Biochar has broad applications in agriculture, where its incorporation into soils is recognized as an effective strategy for improving soil quality, enhancing remediation, sequestering carbon, and mitigating climate change. Although the application of nitrogen fertilizers can enhance nitrogen leaching, integrating biochar may improve nutrient retention and reduce associated losses. However, the effects of biochar on nitrogen and carbon leaching in specific soil types remain unclear. This study investigated the impact of spruce bark biochar (SB550) on the leaching of total nitrogen (TN), nitrate (NO_3_^−^), ammonium (NH_4_^+^), and dissolved organic carbon (DOC) in agricultural soils of eastern Newfoundland. A greenhouse experiment was conducted with *Festulolium* forage grown in a soil–biochar mixture at five biochar rates (0, 2, 5, 8, and 10% *v*/*v*), with and without nitrogen fertilizer (0 and 60 kg N ha^−1^). The results showed that SB550 biochar significantly reduced nutrient and carbon losses (*p* < 0.001). At the 10% biochar rate, leaching of NO_3_^−^, NH_4_^+^, TN, and DOC decreased by 48.6%, 80.4%, 60.0%, and 74.3%, respectively, compared with the control. These findings confirm that the addition of biochar is an effective amendment for minimizing nitrogen and DOC leaching, offering a promising strategy for sustainable nutrient management and environmental protection in this soil type.

## 1. Introduction

Biochar is a carbon-rich organic material produced from forest and agricultural residues such as wood, manure, leaves, and plant waste materials through pyrolysis under limited oxygen conditions. Applying biochar to soil is a promising and effective strategy for improving soil quality and reducing nitrogen (N) and carbon (C) losses [1,2,3,4]. Recently, biochar has attracted more attention as a soil amendment material due to its positive effect on improving soil physical and chemical properties, increasing fertility, and enhancing crop productivity [5,6,7,8]. It also mitigates nutrient leaching [9,10,11], increases soil organic C [5,12,13], cation exchange capacity (CEC), and pH, particularly in acidic soil [5,8,12,14]. Moreover, biochar improves soil structure [15], promotes microbial activity [16,17,18,19], reduces soil bulk density [20,21,22], and enhances water retention [21,23,24].

Despite these advantages, the effectiveness of biochar depends on its feedstock, production conditions, application rate, and soil type. Previous studies have reported wide variability in reductions in N and C leaching following biochar addition. For instance, Steiner et al. (2010) [25] found that mixing poultry litter with 20% biochar reduced total N losses by up to 52%, while Kumar et al. (2016) [26] observed that 20 g corn (*Zea mays*) biochar kg^−1^ soil reduced nitrate leaching by 29% in low-carbon soils. Similarly, Prima et al. (2016) showed that 4% rice (*Oryza sativa*) husk biochar significantly reduced ammonium and nitrate leaching in loamy soils [27]. Pine (*Pinus* spp.) wood biochar at 0.5–10% (*w*/*w*) decreased cumulative nitrate losses by 26–96% and ammonium losses by 12–86% in sandy soils [28]. Most previous biochar studies have been conducted in tropical or temperate regions using arbitrary application rates, with limited attention to cold, humid environments where leaching is intense. The behavior of biochar in highly leached podzolic soils remains poorly understood, despite their inherent acidity, coarse texture, low fertility, and high nutrient losses. Understanding biochar’s performance under such conditions is essential for its effective use in northern agricultural systems.

In Newfoundland and Labrador (NL), Canada, where the government aims to achieve 20% food self-sufficiency [29], agriculture faces major challenges due to the predominance of podzolic soils, formed under high annual precipitation (~1000 mm) [30,31]. These soils are stony, strongly acidic, low-fertility, low-CEC, and have limited soil organic matter (SOM). Such conditions promote nutrient leaching in response to fertilizer use. Nitrogen fertilizer is essential for forage and crop production [32], yet more than 30% of applied inorganic N may be lost through leaching [33,34], posing risks to water quality [35]. Dissolved organic carbon (DOC) is also mobile and contributes to both nutrient depletion and aquatic ecosystem impacts [36,37].

Given NL’s cool, wet climate and nutrient-poor soils, there is a critical need for sustainable amendments that enhance fertility while minimizing losses. High rainfall, coarse soils, and strong acidity in NL create leaching conditions very different from those of other agricultural regions in Canada. These conditions may change how biochar operates in this soil. However, biochar’s effects on nitrogen and carbon leaching in eastern Newfoundland’s podzolic soils have yet to be studied, and thus its effectiveness in this region remains poorly understood. Therefore, this study examines the impact of spruce bark (*Picea* spp.) biochar (SB550) on the leaching of nitrate (NO_3_^−^), ammonium (NH_4_^+^), total nitrogen (TN), and dissolved organic carbon (DOC), to identify the biochar rates that improve nutrient retention under these challenging conditions.

## 2. Materials and Methods

### 2.1. Biochar Production and Characterization

SB550 biochar was selected for this study due to its high nitrate adsorption capacity, which has previously been reported to reach 184 mg g^−1^ [38]. The biochar was obtained from GECA Environnement (Lac-Sergent, QC, Canada) and produced from a spruce bark feedstock at 550 °C (SB550) using Abri-Tech technology [39]. Its physicochemical properties are summarized in Table 1, and detailed information regarding the biochar’s preparation and characteristics is available elsewhere [40,41].

**Figure 1:**
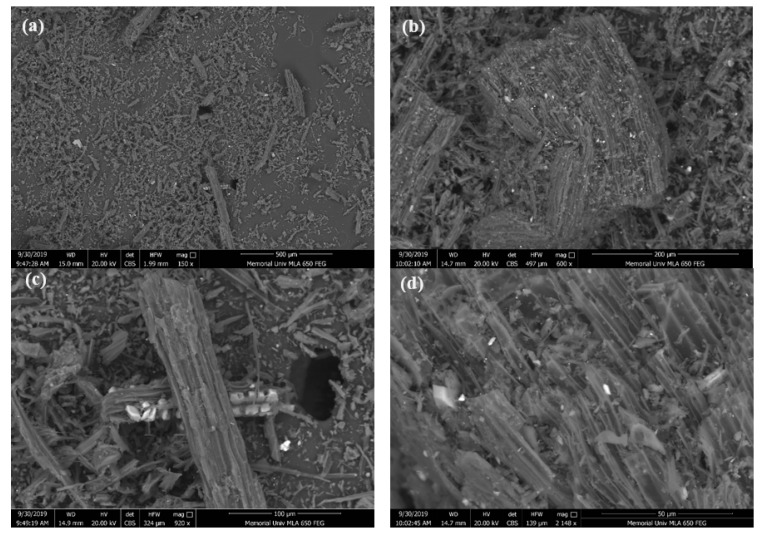
SEM images of spruce bark biochar (SB550) at different scales: (**a**) 500 μm, (**b**) 200 μm, (**c**) 100 μm, and (**d**) 50 μm.

Scanning Electron Microscopy (SEM) was performed using an FEI MLA 650F (FEI Company, Hillsboro, OH, USA) equipped with Bruker XFlash X-ray detectors for Energy-Dispersive Spectroscopy (EDS) and a backscattered electron (BSE) detector for compositional analysis. SEM imaging was used to examine the morphology and porous structure of the SB550 biochar (Figure 1 and Figure S1).

**Figure 2:**
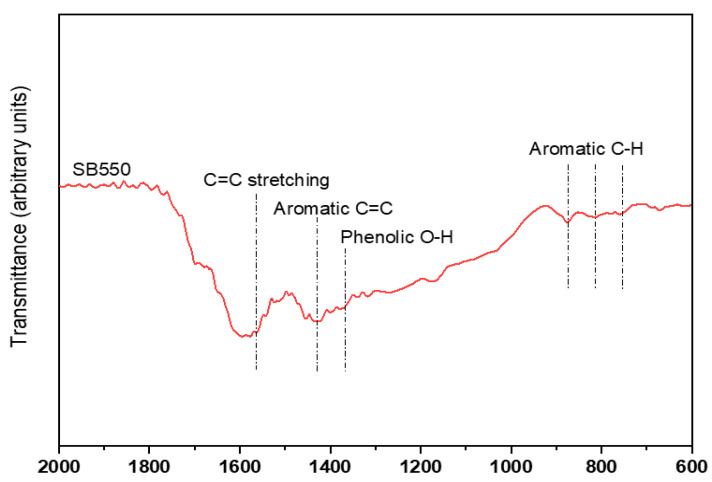
FT-IR spectra of spruce bark biochar (SB550), showing the major surface functional groups present on the biochar.

Fourier Transform Infrared (FT-IR) spectroscopy was conducted using a Nicolet iS50 FT-IR spectrometer (Thermo Fisher Scientific, Waltham, MA, USA) equipped with an attenuated total reflectance (ATR) accessory. Spectra were collected in the mid-infrared range (4000–400 cm^−1^) at 4 cm^−1^ resolution, with an average of 32 scans per sample. Major absorption bands were analyzed to identify surface functional groups, including hydroxyl, carboxyl, and aromatic groups, which are known to play key roles in nutrient retention, soil interactions, and biochar stability (Figure 2).

### 2.2. Experimental Set-Up and Design

The experiment was conducted in a greenhouse at the Memorial University Botanical Garden (St. John’s, Canada) between December 2018 and April 2019. A *Festulolium* forage crop was grown in plastic pots (30.48 cm in diameter and 27.5 cm in height; Figure S1), each filled with 15 kg of soil. The plants were cultivated under a controlled photoperiod of 16 h light and 8 h dark, at a temperature range of 18–20 °C. *Festulolium* is a hybrid forage grass developed by crossing Meadow or Tall Fescue with Perennial or Italian Ryegrass [42]. The soil used in the experiment was classified as a silt loam within the Podzol order according to the Canadian System of Soil Classification (CSSC) [43]. It was collected from the agricultural research site at St. John’s Research and Development Centre. The experiment followed a completely randomized design (CRD) conducted in a greenhouse, with each treatment replicated three times (*n* = 3). Five biochar application rates were tested: 0% (control), 2%, 5%, 8%, and 10% (*v*/*v*) (Figure S2). Two nitrogen fertilizer levels were applied using urea (46% N) at 0 and 60 kg N ha^−1^. Each biochar–nitrogen combination underwent further testing in two cropping conditions, with and without *Festulolium* forage (crop and no-crop treatments), resulting in a total of 20 treatment combinations (5 biochar rates × 2 N levels × 2 crop conditions). In total, 60 pots were established and randomly arranged in the greenhouse (Figure S3).

### 2.3. Soil, Biochar, and Nitrogen Fertilizer

The soil was collected from the agricultural research site of Agriculture and Agri-Food Canada at St. John’s Research and Development Centre (47°31′ N, 52°47′ W at an elevation of 115 m above sea level). Soil samples were taken from the top 20 cm and dried in a forced-air drying room at 35 °C for 72 h. Biochar was thoroughly dry-mixed with portions of the soil at rates of 0%, 2%, 5%, 8%, and 10% [*v*/*v*]. The resulting mixtures were then uniformly incorporated into the top 10 cm of soil above the remaining portion to ensure that they were evenly distributed throughout the surface layer rather than being applied as a separate layer. Two nitrogen fertilizers containing urea (46% N) were applied to the top 2.5–3.0 cm of soil at rates of 0 and 60 kg N ha^−1^. Finally, *Festulolium* seeds were uniformly sown in the top 0.5 cm of soil at the recommended field seeding rate of 39 kg ha^−1^, adjusted to the pot surface area. A preliminary experiment was conducted to estimate the amount of water required for each pot to avoid excessive leaching and to determine the soil field capacity (FC). The soil FC was measured as 35% (g water g^−1^ oven-dry soil) using the gravimetric oven-drying method. To this end, wet soil samples were collected 24 h after water addition, and soil moisture content was determined using the standard drying method, in which 5 g soil samples were dried at 105 °C for 48 h. The soil moisture content of the pots was monitored every 3–4 days using the GS3 sensor for volumetric water content (VWC), temperature, electrical conductivity of water (ECw), and the ProCheck Sensor Read-Out and Storage System (HOSKIN SCIENTIFIC LTD., Oakville, ON, Canada). All pots were watered to field capacity, ensuring that the soil moisture content was maintained at above 20% of the total available water. This greenhouse study provides valuable preliminary insights; however, long-term field experiments are needed to confirm biochar’s effects on soil properties and plant performance under natural conditions. Such research will be essential for developing sustainable nutrient management strategies and improving soil health in Newfoundland’s challenging agricultural environments.

### 2.4. Leachate Sampling and Analysis

Leachate samples were collected from trays installed beneath the pots every three weeks, within 24 h after irrigation, using 60 mL syringes. The full volume of leachate drained from each pot during each collection event was measured and recorded immediately after sampling to ensure consistent quantification across treatments. The collected leachate was transferred into 250 mL polyethylene bottles and stored at −20 °C until analysis. Each parameter was analyzed using the corresponding analytical method. The pH and electrical conductivity (EC) of the leachate were measured using a Hach HQ40d portable multi-parameter meter (Hach Company, Loveland, CO, USA). Subsamples were filtered through 0.45 μm membrane filters before undergoing further analysis. The nitrate (NO_3_^−^) and ammonium (NH_4_^+^) concentrations were determined using a Seal Analytical Continuous Flow Analyzer (AA3 HR, Seal Analytical Inc., Norderstedt, Germany) [44,45]. The total nitrogen (TN) in leachate and the dissolved organic carbon (DOC) content were measured directly using a TOC/TN analyzer (TOC-LCPH, Shimadzu Corporation, Kyoto, Japan). The leachate results are expressed in mg L^−1^ and reported on a concentration basis; flux-based estimates of nutrient losses were not calculated, which should be considered as a limitation when interpreting the magnitude of leaching.

### 2.5. Statistical Analysis

Statistical analyses were conducted using a general linear model in Minitab 19 software [46]. The effects of biochar (B), nitrogen (N), crop (C), and their interactions were evaluated using analysis of variance (ANOVA) at a significance level of α ≤ 0.05, based on the completely randomized design (CRD). Prior to the ANOVA, the assumptions of normality and homogeneity of variance were tested and satisfied using the Shapiro–Wilk test for normality and Levene’s test for homogeneity of variance, and residual plots were inspected. When significant treatment effects were observed, mean separations were performed using Tukey’s test at the 95% confidence level (*p* < 0.05). A three-way ANOVA was applied according to the linear model Equation (S1) [47].

## 3. Results

### 3.1. Leachate pH and EC

**Figure 3:**
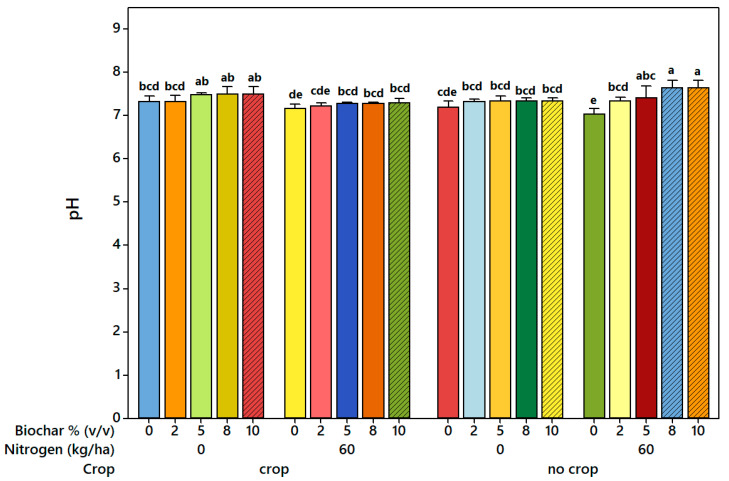
Effect of biochar application, nitrogen levels, and crop type on soil leachate pH. Each bar represents the mean (*n* = 3), and vertical error bars indicate the standard error of the mean (SEM). Different letters (a–e) above the bars indicate significant differences among treatments based on Tukey’s HSD test (*p* < 0.05).

The statistical analysis of leachate pH showed that biochar application had a significant effect (*p* < 0.001) (Table S1). Tukey’s test confirmed that all biochar rates (2, 5, 8, and 10% [*v*/*v*]) significantly increased leachate pH compared to the control (0% [*v*/*v*]) (Figure 3). pH increased by approximately 0.1, 0.2, and 0.3 units at 2, 5, and 8–10% biochar, respectively, without nitrogen addition, and by 0.2, 0.3, and 0.4 units at the same biochar rates when 60 kg N ha^−1^ was applied. The interaction between biochar and nitrogen was significant (*p* = 0.011), with the highest pH occurring in the sample containing 8–10% biochar combined with nitrogen. Biochar and crop interaction was also significant (*p* = 0.004), with stronger increases in the no-crop treatments, and the three-way interaction showed that pH consistently increased with rising biochar rates and nitrogen application (Figure 3). Although modest, these pH increases are meaningful for strongly acidic, low-buffered podzolic soils, potentially enhancing nutrient availability, reducing aluminum toxicity, and supporting microbial activity.

**Figure 4:**
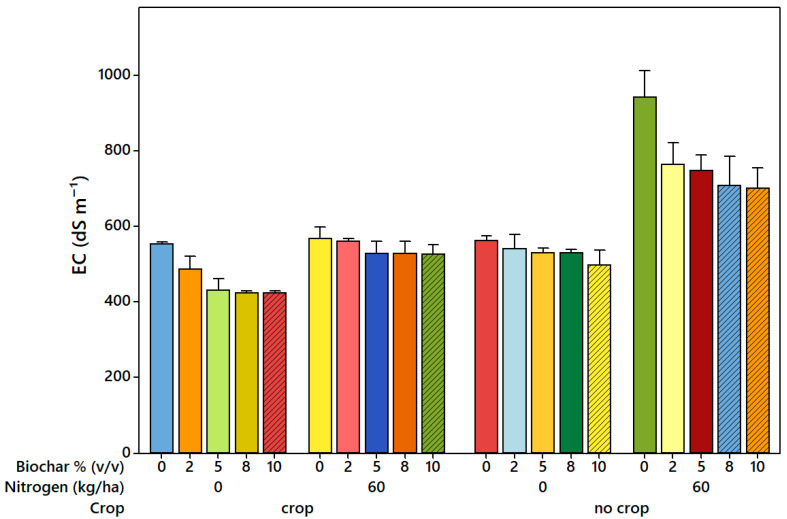
Effect of biochar application, nitrogen levels, and crop type on soil leachate electrical conductivity (EC, dS m^−1^). Each bar represents the mean (*n* = 3), and vertical error bars indicate the standard error of the mean (SEM).

The ANOVA of leachate EC revealed a significant reduction with increasing biochar application rate, nitrogen level, and crop treatment (*p* < 0.001) (Table S2). Relative to the controls, biochar consistently decreased EC values across all treatments. In treatments without nitrogen, EC declined from approximately 0.55 dS m^−1^ in the control to 0.47, 0.44, and 0.42 dS m^−1^ at 2%, 5%, and 10% biochar, respectively. When nitrogen was added (60 kg N ha^−1^), the EC ranged between 0.50 and 0.45 dS m^−1^, showing slight but consistent reductions as the biochar rate increased. Under crop conditions, EC values were generally lower (≈0.40–0.55 dS m^−1^) compared to those observed for the no-crop treatments. The highest EC values (up to 1.0 dS m^−1^) occurred in the no-crop control, whereas biochar addition (2–10%) markedly reduced EC to ≈0.6 dS m^−1^. Overall, leachate EC declined progressively with increasing biochar application rate, particularly in the no-crop and non-fertilized treatments (Figure 4).

### 3.2. Leachate Nitrate and Ammonium

**Figure 5:**
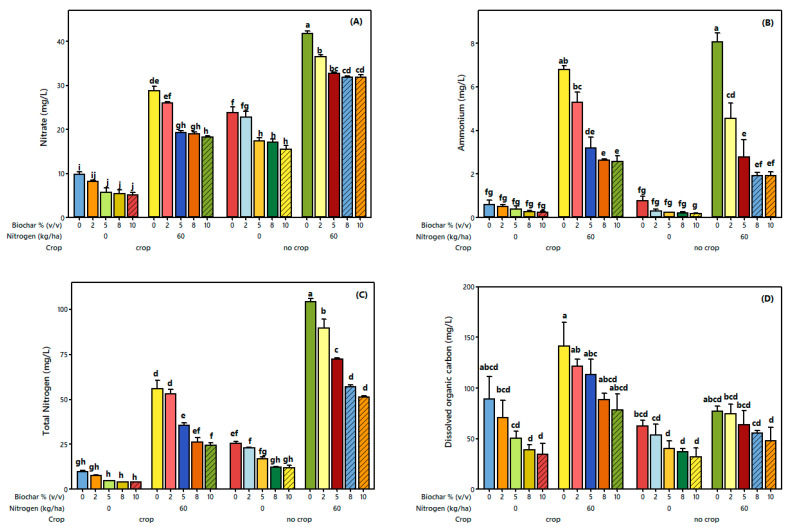
The effects of biochar application, nitrogen levels, and crop type on soil leachate properties, including (**A**) nitrate (NO_3_^−^), (**B**) ammonium (NH_4_^+^), (**C**) total nitrogen (TN), and (**D**) dissolved organic carbon (DOC). Values are presented as the mean ± SE (*n* = 3), and vertical error bars indicate the standard error. Different letters (a–j) above the bars indicate significant differences among treatments based on Tukey’s HSD test (*p* < 0.05).

The ANOVA of leachate nitrate (Table S3) indicated that biochar, nitrogen, and crop treatments significantly influenced nitrate leaching. In crop treatments without nitrogen, nitrate concentrations decreased progressively with increasing biochar rate by 16.5% at 2%, 41.8% at 5%, 45.3% at 8%, and 48.6% at 10% (Table S3 and Figure 5A). For crops treated with nitrogen, we observed reductions of 9.9%, 33.0%, 34.1%, and 36.9% at 2%, 5%, 8%, and 10% biochar, respectively (Table S3 and Figure 5A). In treatments without crop and without nitrogen, nitrate decreased by 4.2%, 27.4%, 28.4%, and 34.8% at 2%, 5%, 8%, and 10% biochar, respectively (*p* < 0.001) (Table S3 and Figure 5A). In treatments without crop but with added nitrogen, we observed reductions of 12.8%, 21.7%, 24.0%, and 23.9% at 2%, 5%, 8%, and 10% biochar, respectively (*p* < 0.001) (Table S3 and Figure 5A). Overall, nitrate leaching decreased consistently with increasing biochar rates across all treatments, with the strongest reductions observed in the crop without nitrogen, while moderate reductions occurred in treatments with nitrogen or without crops, demonstrating biochar’s effectiveness in minimizing nitrate losses from fertilized cropping systems.

Furthermore, the ANOVA results for leachate ammonium (Table S4) showed that both biochar and nitrogen treatments significantly affected ammonium leaching (*p* < 0.001). Nitrogen addition caused a marked increase in ammonium concentrations, rising from 0.8 mg L^−1^ in the control (0 kg N ha^−1^) to 8.0 mg L^−1^ at 60 kg N ha^−1^, an increase of 7.2 mg L^−1^. Biochar application significantly reduced ammonium leaching across all treatments (*p* < 0.001). In crop treatments without nitrogen, ammonium concentrations decreased by 16.1%, 35.0%, 56.7%, and 59.4% at 2%, 5%, 8%, and 10% biochar, respectively. In response to crop treatments with nitrogen, we observed reductions of 22.2%, 53.0%, 61.5%, and 62.2% at the corresponding biochar levels. In treatments without crop and without nitrogen, biochar decreased ammonium leaching by 62.1%, 71.3%, 74.4%, and 80.4% at 2%, 5%, 8%, and 10%, respectively. Similarly, in non-cropped treatments with nitrogen, reductions of 43.7%, 65.9%, 76.5%, and 76.5% were observed at increasing biochar levels. Overall, ammonium leaching declined progressively with increasing biochar rates, confirming the effectiveness of biochar in mitigating nitrogen losses under controlled greenhouse conditions (Table S4; Figure 5B).

### 3.3. Leachate Total Nitrogen and Dissolved Organic Carbon

The ANOVA results for leachate total nitrogen (TN) (Table S5) indicated that biochar, nitrogen, and crop treatments significantly influenced TN leaching (*p* < 0.001). Nitrogen addition markedly increased TN concentrations, which rose by 375% at the 60 kg N ha^−1^ level compared to the control (0 kg N ha^−1^) under cropped conditions, and by 106% under no-crop conditions (*p* < 0.001) (Table S5; Figure 5C). Biochar application significantly reduced TN leaching across all treatments (*p* < 0.001). In crop treatments without nitrogen, we observed reductions of 22.0%, 52.9%, 60.0%, and 60.0% at 2%, 5%, 8%, and 10% biochar, respectively. Under crop treatments with nitrogen, we noted reductions of 5.3%, 36.8%, 53.1%, and 56.2% at the corresponding biochar levels. In non-cropped treatments without nitrogen, TN leaching decreased by 9.3%, 33.2%, 51.3%, and 52.8% at 2%, 5%, 8%, and 10% biochar, respectively. Similarly, in non-cropped treatments with nitrogen, we noted reductions of 14.0%, 30.5%, 45.1%, and 50.5%. Overall, TN leaching decreased progressively with increasing biochar rates across all treatments, demonstrating the strong capacity of biochar to mitigate nitrogen losses under controlled greenhouse conditions. The total nitrogen in leachate was calculated as the sum of nitrate (NO_3_^−^) and ammonium (NH_4_^+^) concentrations.

Moreover, the ANOVA of leachate dissolved organic carbon (DOC) (Table S6) indicated that biochar, nitrogen, and crop treatments significantly affected DOC leaching (*p* < 0.001 for all factors). Nitrogen addition increased DOC leaching by 69.13% at the 60 kg N ha^−1^ level relative to the control (0 kg N ha^−1^) (Table S6 and Figure 5D). In contrast, the absence of crops significantly reduced DOC leaching by 34.21% compared to crop treatments (*p* < 0.001) (Table S6 and Figure 5D). Biochar application significantly influenced DOC leaching (*p* < 0.001), with reductions of 13.43%, 27.70%, 40.33%, and 74.26% at biochar rates of 2%, 5%, 8%, and 10% [*v*/*v*], respectively, compared to the control (0% [*v*/*v*]). In crop treatments without nitrogen, DOC decreased progressively from 17.3% at 2% biochar to 33.6% at 5%, 44.0% at 8%, and 62.4% at 10%. For crop treatments with nitrogen, reductions ranged from 19.5% at 2% to 38.0% at 5%, 48.2% at 8%, and 51.6% at 10%. In treatments without crop and without nitrogen, DOC reductions ranged from 11.0% at 2% to 56.0% at 10%, while in treatments without crop and with nitrogen, reductions ranged from 20.0% to 57.6%. Overall, DOC leaching decreased with increasing biochar rates across all treatments, highlighting the role of biochar in mitigating carbon losses under controlled greenhouse conditions. Overall, DOC leaching decreased with increasing biochar rates across all treatments, highlighting the strong role of biochar in mitigating carbon losses under controlled greenhouse conditions. Since the biochar was applied without pre-washing, the substantial reductions in DOC (up to 74%) may partly result from the leaching of biochar-derived soluble organic fractions (Table S6; Figure 5D).

## 4. Discussion

### 4.1. Effect of Biochar on Soil Leachate pH and EC

The soil leachate pH in this study ranged from 7.03 in the control (0% [*v*/*v*] biochar) to 7.63 at 10% [*v*/*v*] biochar (Figure 3), with a significant, dose-dependent increase (*p* < 0.001; Table S1). These results are consistent with previous studies by Angst et al. (2013), and Bradley et al. (2015), which also reported rising leachate pH with higher biochar rates [48,49]. The increase in pH was primarily attributed to the high alkalinity of the biochar used (pH = 9.9; Table 1), but additional factors contributed. Carbonates and surface functional groups (oxides and organic anions) help neutralize soil acidity [50], while the reduction in exchangeable Al^3+^ is a major source of acidity in soils [51]. It occurs through hydroxyl-aluminum polymerization, Al hydroxide precipitation, and displacement by base cations (Ca^2+^, Mg^2+^, K^+^, Na^+^) released from biochar [52,53,54]. Formation of organo–Al complexes with biochar functional groups further supports long-term acid amelioration [55,56]. Overall, biochar increases soil pH both directly through alkalinity and indirectly via cation exchange processes. These effects were moderate in our study, likely because the low base saturation of the Podzol soils limits the persistence of biochar’s liming potential, highlighting the influence of baseline soil conditions on the magnitude of biochar’s effect.

Furthermore, soil leachate pH was significantly influenced by the interaction of nitrogen and crop treatments, with the combination of 60 kg N ha^−1^ and no-crop conditions showing higher pH values than the same nitrogen rate under crop treatments (*p* < 0.004) (Table S1). Nitrogen fertilization, particularly urea, can temporarily increase soil pH via hydrolysis that produces OH^−^ and NH_4_^+^, but this effect is moderate and can be offset by subsequent nitrification, which releases H^+^ ions [57,58]. Biological mechanisms further modulate soil pH: plant roots release protons when absorbing more cations than anions, secrete organic acids, and influence microbial activity, all of which tend to acidify the rhizosphere [59]. Microbial processes, including nitrification and organic matter decomposition, also contribute to local pH changes [55,56,57]. Biochar’s inherent alkalinity and high cation exchange capacity help buffer these acidifying effects, moderating pH fluctuations [54]. Overall, the combined chemical, biological, and biochar-mediated processes result in a moderate but meaningful increase in soil pH, highlighting the interplay of abiotic and biotic factors in regulating soil acidity.

Biochar’s effect on soil leachate electrical conductivity (EC) demonstrated a clear decreasing trend with increasing biochar application rates (*p* < 0.001) (Figure 4 and Table S2). Specifically, the leachate EC in the control treatment (0% [*v*/*v*]) was significantly higher than in treatments containing 2%, 5%, 8%, and 10% biochar, with reductions of 12.0%, 17.7%, 23.4%, and 23.4%, respectively, relative to the control (Figure 4). These findings align with previous studies indicating that biochar can reduce soluble salt concentrations in soil leachates by enhancing the adsorption capacity for cations, making them less prone to leaching [60,61]. The reduction in leachate EC at higher biochar rates is particularly pronounced due to both chemical and physical mechanisms. Chemically, the increased surface area and functional groups of biochar (e.g., carboxyl, hydroxyl, and phenolic groups) enhance cation retention [62]. Physically, biochar improved soil structure and porosity with increasing application rates, enhancing water infiltration and flow paths. Despite faster water movement at higher rates, its high surface area and nutrient adsorption capacity retained nitrogen and dissolved organic carbon, thereby reducing leaching. In contrast, control or low-biochar soils retained water longer but had limited nutrient retention [28,63]. Nitrogen and crop treatments further influenced leachate EC (Table S2). Maximum EC occurred at low biochar rates with high nitrogen in no-crop treatments (Figure 4), indicating that biochar adsorbs ammonium and nitrate, slowing their leaching [62]. In contrast, lower EC in response to crop treatments reflects biological mechanisms, and increased nutrient uptake by plants reduces soluble ion concentrations [64]. Overall, leachate EC is modulated by the interplay of biochar’s chemical adsorption, physical effects on soil water movement, and biological nutrient uptake.

### 4.2. Effect of Biochar on Soil Leachate Total Nitrogen, Nitrate, and Ammonium

Nitrogen leaching is strongly influenced by fertilizer management, crop and residue management, and water practices [65,66,67]. In this study, the total nitrogen (TN) in leachate increased with the N application rate, with the 60 kg N ha^−1^ treatment showing significantly higher TN than the control (0 kg N ha^−1^; *p* < 0.001) (Table S5). Nitrate was the dominant N form in leachate, showing a stronger correlation (*R*^2^ = 0.964; *p* < 0.001) than ammonium (*R*^2^ = 0.786; *p* < 0.001) (Table S7), consistent with previous reports under upland crops [66,67].

Application of spruce bark biochar significantly reduced nitrate concentrations in leachate (*p* < 0.001; Table S3 and Figure 5). The spruce bark biochar used exhibited a high nitrate adsorption capacity (maximum 184 mg g^−1^) [38]. FT-IR spectra (Figure 2) revealed distinct absorption bands associated with aromatic C=C stretching, phenolic O–H bending, and carboxyl (–COOH) groups—functional groups known to facilitate nitrate adsorption through electrostatic attraction and hydrogen bonding. These surface functionalities, together with the biochar’s porous structure and high surface area, increase the number of active adsorption sites and physically entrap nitrate ions. Moreover, spruce bark biochar indirectly reduces nitrate mobility by improving soil structure and water retention. These groups facilitate nitrate adsorption, reducing leaching losses [27,68]. Nitrate leaching decreased progressively with increasing spruce bark biochar rates, consistent with previous studies demonstrating biochar’s ability to minimize nitrate concentrations in leachates [37,69]. Mechanistically, nitrate reduction can occur through the (i) mass flow of nitrate ions into biochar pores and subsequent incorporation onto its surface [70,71]; (ii) electrostatic interactions between negatively charged nitrate ions and positively charged surface groups or cationic salts [71,72]; (iii) suppression of nitrification through ammonium adsorption, which limits substrate availability [26,69,73]; and (iv) microbial nitrogen immobilization or transformation into organic N pools [49,74]. Similar mechanisms operate for ammonium, where spruce bark biochar adsorbs NH_4_^+^, inhibits its mineralization and/or nitrification, and immobilizes inorganic N, thereby reducing leaching [73,75,76,77,78]. Overall, spruce bark biochar effectively mitigates nitrate and ammonium leaching through an integrated set of physical adsorption, chemical surface interactions, and biological processes, supporting its role as a sustainable soil amendment that enhances nitrogen retention and fertilizer use efficiency in agricultural systems.

Biochar can act as an ammonium-retaining soil amendment, as reported in several studies [62,69,79]. When urea is applied to soil, urease enzymes rapidly hydrolyze it to ammonium bicarbonate, which is subsequently converted to nitrate through nitrification. Biochar application, however, has been shown to reduce both ammonium and nitrate leaching [69], which is consistent with the findings presented in the current study. Our results showed that ammonium reduction was significantly affected by biochar application and followed a similar trend to nitrate reduction (*p* < 0.001) (Table S4 and Figure 5B). The reduction in ammonium was directly proportional to the biochar rate, with the 10% [*v*/*v*] treatment showing the highest ammonium retention. Furthermore, correlation analysis between ammonium and nitrate in the leachate showed a significant positive Pearson correlation (*r* = 0.691, *p* < 0.001) (Table S7), indicating that the two forms of nitrogen respond to biochar addition in a related manner. Notably, the percentage reduction in ammonium leaching was higher than that of nitrate, ranging from 34.65% to 70.04% across the 2% to 10% biochar rates, compared to 11.61% to 47.75% for nitrate. This pattern reflects a fundamental chemical distinction between the two ions: ammonium (NH_4_^+^) is a cation that is readily attracted to and retained by negatively charged soil and biochar surfaces, whereas nitrate (NO_3_^−^) is an anion that is more mobile and prone to leaching. Accordingly, the high cation exchange capacity (CEC) and porous structure of biochar preferentially retain ammonium ions while also slowing nitrate movement through adsorption and microbial immobilization. The higher reductions in ammonium losses can be explained by several interrelated mechanisms. First, the low nitrification rate in biochar-amended soils may have contributed to reduced ammonium losses. Biochar has been shown to inhibit nitrification processes by altering soil microbial communities and releasing inhibitory compounds, such as labile organic carbon or phenolic groups, which suppress nitrifying bacteria [73,79]. This finding supports the idea that the biochar used in this study effectively limited nitrification, although the degree of inhibition may vary depending on feedstock type and pyrolysis conditions [79]. Second, ammonium retention via biochar adsorption likely played a significant role. The high CEC of biochar, attributed to negatively charged and oxygen-containing functional groups (e.g., carboxyl, carbonyl, and phenol groups) on its surface, provides abundant sites for ammonium adsorption [80,81,82]. In this study, the biochar had a CEC of 28.8 cmol kg^−1^ (Table 1), and its incorporation increased the soil CEC from 14.6 to 18.6 cmol kg^−1^ (Table S8), which likely enhanced the soil’s ammonium-holding capacity. These results are consistent with earlier studies, which reported that higher soil CEC induced by biochar reduces ammonium leaching by improving nutrient retention [28,83]. Additionally, the physical characteristics of biochar contribute to ammonium adsorption. Several studies have indicated that the large surface area and porous structure of biochar can trap ammonium ions, further decreasing their mobility [27,84]. Saleh et al. (2012) proposed that the physical entrapment of ammonium within biochar pores provides an additional adsorption mechanism [85]. Considering that the ionic diameter of ammonium is approximately 286 pm [86] and the biochar pore sizes range from 0.0001 μm to 1000 μm [5], our results support the theory that physical entanglement within biochar pores may significantly contribute to ammonium retention. Overall, the findings align with the previous literature suggesting that biochar reduces nitrogen leaching through a combination of biochemical inhibition of nitrification, enhanced soil CEC, surface adsorption, and physical entrapment within porous structures. Although biochar can adsorb nitrogen and temporarily reduce its immediate availability to plants, numerous studies provide direct experimental evidence that this immobilized nitrogen is gradually released, often enhancing nitrogen use efficiency (NUE) over time [87,88]. Together, these mechanisms explain why biochar effectively mitigates total nitrogen and ammonium losses, particularly under varying nitrogen application rates and crop treatments.

### 4.3. Effect of Biochar on Soil Leachate Total Nitrogen and Dissolved Organic Carbon

Dissolved organic carbon (DOC) represents a small but highly active fraction of soil organic carbon, primarily derived from biological transformations of plant and microbial residues [89]. Although it only accounts for about 1% of the total soil carbon pool, DOC plays a critical role in nutrient cycling and soil water interactions due to its mobility and chemical reactivity [90]. In the present study, biochar application significantly decreased the DOC concentrations in the leachate (*p* < 0.001; Table S6 and Figure 5D), consistent with previous studies’ findings that biochar mitigates DOC losses in agricultural soils [37,91,92,93]. This reduction can be attributed to several mechanisms. (i) Alteration of soil physicochemical properties: biochar’s high specific surface area enhances adsorption capacities, while its liming effect increases soil pH and CEC in acidic soils, both of which promote DOC retention [16,86,94]. (ii) Stabilization of biochar in soil: strong interactions between biochar particles and soil matrices reduce microbial and chemical decomposition of labile organic fractions, thereby limiting DOC mobilization [92,93]. The interaction between nitrogen application and crop presence observed in this study provides additional insight into DOC dynamics. In 60 kg N ha^−1^ treatments with crops, the DOC concentrations in leachates increased compared with the no-crop treatments. Conversely, nitrogen fertilization increased DOC concentrations, particularly in response to crop treatments. This pattern likely reflects enhanced microbial priming and root exudation under elevated nitrogen availability, which stimulates microbial decomposition of native soil organic matter and releases soluble organic compounds [91,95]. Nitrogen-induced stimulation of root exudation, including the release of low-molecular-weight organic acids, sugars, and amino acids, can enrich the DOC pool by providing substrates for microbial metabolism and by mobilizing organic carbon from mineral surfaces. Nitrogen-induced exudation of low-molecular-weight organic acids, sugars, and amino acids may further enrich the DOC pool by providing microbial substrates and mobilizing carbon bound to mineral surfaces. The higher DOC observed in cropped soils compared to no-crop treatments underscores the contribution of root-derived carbon inputs, consistent with the findings of Royer et al. (2007), Chibuike et al. (2019), and Salazar et al. (2019) [90,95,96].

DOC behavior in this controlled setting, however, may differ under field conditions, where rainfall variability, temperature fluctuations, and microbial turnover dynamically regulate DOC production and transport. Natural wet–dry cycles and preferential flow pathways can generate transient DOC pulses that partially offset biochar’s adsorptive effects.

Importantly, this study is among the first to investigate the coupled biochar–nitrogen–carbon interactions in maritime podzolic soils, a cold, coarse-textured environment with distinct organic matter dynamics compared to typical agricultural soils. The spruce bark biochar (SB550, 550 °C) used here, which is rich in aromatic and oxygen-containing functional groups, effectively enhanced DOC adsorption and reduced leaching losses. By integrating biochar application, nitrogen fertilization, and a forage grass system, this work provides novel insights into how biochar can regulate DOC and nitrogen fluxes in cool, nitrogen-limited ecosystems. These findings have broader implications for long-term carbon sequestration and sustainable nutrient management, supporting the role of biochar as a multifunctional amendment for enhancing soil carbon stability and minimizing nutrient leaching in temperate agroecosystems.

The present study demonstrated that biochar application significantly reduced total nitrogen (TN) and dissolved organic carbon (DOC) leaching across all treatments, while nitrogen addition markedly increased both TN and DOC concentrations (*p* < 0.001) (Tables S5 and S6; Figure 5C,D). A significant positive Poisson correlation coefficient was observed between DOC and TN (Table S7), indicating a link in the behaviors of carbon and nitrogen dynamics in the leachate. The DOC concentration followed the same trend as nitrogen fertilization, suggesting that biological mechanisms largely mediate DOC production and consumption under nitrogen application in nitrogen-limited ecosystems [97]. Several biological mechanisms may explain the observed increases in DOC under nitrogen addition: (i) increased production of organic substrates in the soil, providing more precursors for DOC formation [81], (ii) enhanced enzymatic activity, which promotes the release of DOC into the soil solution [98,99]; and (iii) reduced mineralization of organic carbon, leading to slower DOC decomposition and accumulation in deeper soil layers [100]. These mechanisms are consistent with our findings, which show that nitrogen fertilizer treatments increased DOC leaching, despite theoretical expectations of nitrogen-driven DOC mineralization. Previous studies have reported contrasting effects. For example, Tiefenbacher et al. (2020) suggested that nitrogen-based fertilizers reduce leachate DOC concentrations by accelerating DOC mineralization, thereby reducing DOC losses [89]. Nitrate can stimulate the conversion of organic carbon to CO_2_, decreasing DOC leaching. However, the present results showed that DOC increased under nitrogen fertilizer treatments, indicating that in these systems, the biological mechanisms described above likely outweigh DOC mineralization effects, leading to its accumulation in the leachate. These findings support the theoretical framework that biochar mitigates nutrient leaching by improving soil retention and microbial regulation of carbon and nitrogen cycles. The reduction in TN and DOC with increasing biochar rates aligns with previous studies demonstrating biochar’s role in adsorbing nutrients, enhancing soil cation exchange capacity, and promoting microbial immobilization of nitrogen and organic carbon. Thus, this study highlights that biochar not only reduces nutrient losses but also interacts with nitrogen fertilization to modulate carbon dynamics in leachate, providing insight into the mechanisms underlying biochar’s effects on soil properties.

## 5. Conclusions

This study demonstrates that spruce bark biochar (SB550) substantially improves soil leachate chemistry in podzolic soil by enhancing nutrient retention and moderating soil solution properties. At the highest application rate, SB550 reduced the leaching of nitrate, ammonium, total nitrogen, and dissolved organic carbon by 48.6%, 80.4%, 60.0%, and 74.3%, respectively, indicating a strong capacity to limit both inorganic nitrogen and carbon losses. These reductions occurred alongside a 25.7% decline in electrical conductivity, reflecting decreased soluble salt concentrations, and an increase in leachate pH of up to 8.5%, demonstrating the amendment’s liming effect. Collectively, these responses show that the combined liming and adsorptive properties of SB550 improve nutrient retention, enhance nitrogen use efficiency, and mitigate environmentally detrimental leaching processes in coarse-textured podzolic soils. The simultaneous reduction in EC and retention of mobile nutrient forms underscores biochar’s potential to stabilize soil solution chemistry and improve the sustainability of nutrient management strategies. Although the greenhouse conditions provide clear evidence of SB550’s effectiveness, long-term field trials are necessary to validate these outcomes with regard to natural environmental variability and to determine the persistence of these benefits across growing seasons and management systems.

## Figures and Tables

**Table 1:** Properties of SB550 biochar: moisture content, pH, cation exchange capacity (CEC), total nitrogen, total carbon, total phosphorus, total potassium, total calcium, total magnesium, and soluble nutrients.

Analysis	SB550
Moisture Content (%)	<1
pH	9.9
Total Nitrogen, N (%)	0.95
Total Carbon, C (%)	77.2
Total Phosphorous, P (%)	0.29
Total Potassium, K (%)	1.33
Total Calcium, Ca (%)	1.75
Total Magnesium, Mg (%)	0.24
Soluble Salts (dS m^−1^)	0.7
CEC (cmol kg^−1^)	28.5
Basic properties of SB550 biochar.	

## Data Availability

Data are contained within the article and Supplementary Materials.

## Supplementary Materials

The following supporting information can be downloaded at: https://www.mdpi.com/article/10.3390/plants14233687/s1, Figure S1: Soil column in the experiment pots used for soil leachate collection; Figure S2: Soil-biochar mixtures prepared at five application rates, which were added as percentages based on the soil volume [*v*/*v*] (0, 2, 5, 8 and 10%) in the top 10 cm of the soil; Figure S3: Experimental layout, showing biochar, nitrogen, and crop levels and the treatment descriptions within a completely randomized design (CRD); Table S1: Analysis of variance (ANOVA) of estimated soil leachate pH in response to the main effects of biochar, nitrogen levels, crop, and interactions; Table S2: Analysis of variance (ANOVA) of estimated soil leachate EC (μs/cm) in response to the main effects of biochar, nitrogen levels, crop, and interactions; Table S3: Analysis of variance (ANOVA) of estimated soil leachate nitrate (mg/L) in response to the main effects of biochar, nitrogen levels, crop, and interactions; Table S4: Analysis of variance (ANOVA) of estimated soil leachate ammonium (mg/L) in response to the main effects of biochar, nitrogen levels, crop, and interactions; Table S5: Analysis of variance (ANOVA) of estimated soil leachate total nitrogen (mg/L) in response to the main effects of biochar, nitrogen levels, crop, and interactions; Table S6: Analysis of variance (ANOVA) of estimated soil leachate dissolved organic carbon (mg/L) in response to the main effects of biochar, nitrogen levels, crop, and interactions; Table S7: Correlation coefficients between the leached parameters, including nitrate, ammonium, total nitrogen, total dissolved carbon, pH, and EC (*n* = 60); Table S8: Mean values of Final soil pH, % N, % C, % SOM, and CEC in response to biochar, nitrogen fertilizer and crops treatments used in the experiment.
