# Supplementary material for: Application of Spruce Bark Biochar Minimizes Nitrogen and Carbon Leaching from an Eastern Newfoundland Podzolic Soil
Source: Plants (Basel). 2025 Dec 3;14(23):3687. doi: 10.3390/plants14233687 (PMC12694051; doi:10.3390/plants14233687)
Supplement: Supplementary file 1 [file plants-14-03687-s001.zip › plants-3992618-supplementary.pdf]

Supplementary materials

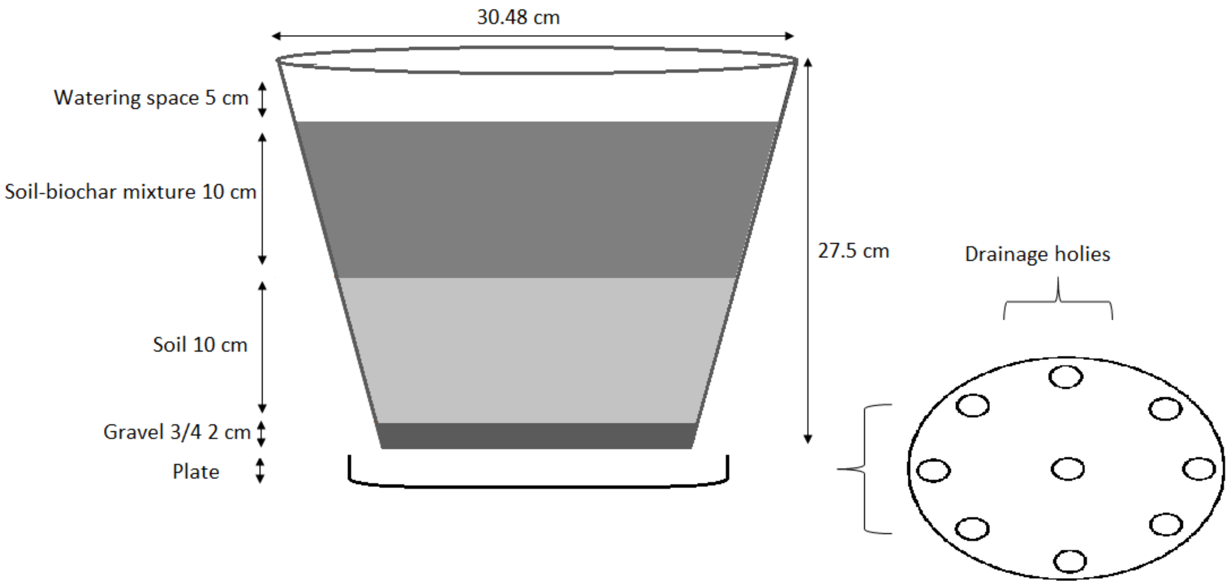

Figure S1. Soil column in the experiment pots used for soil leachate collection

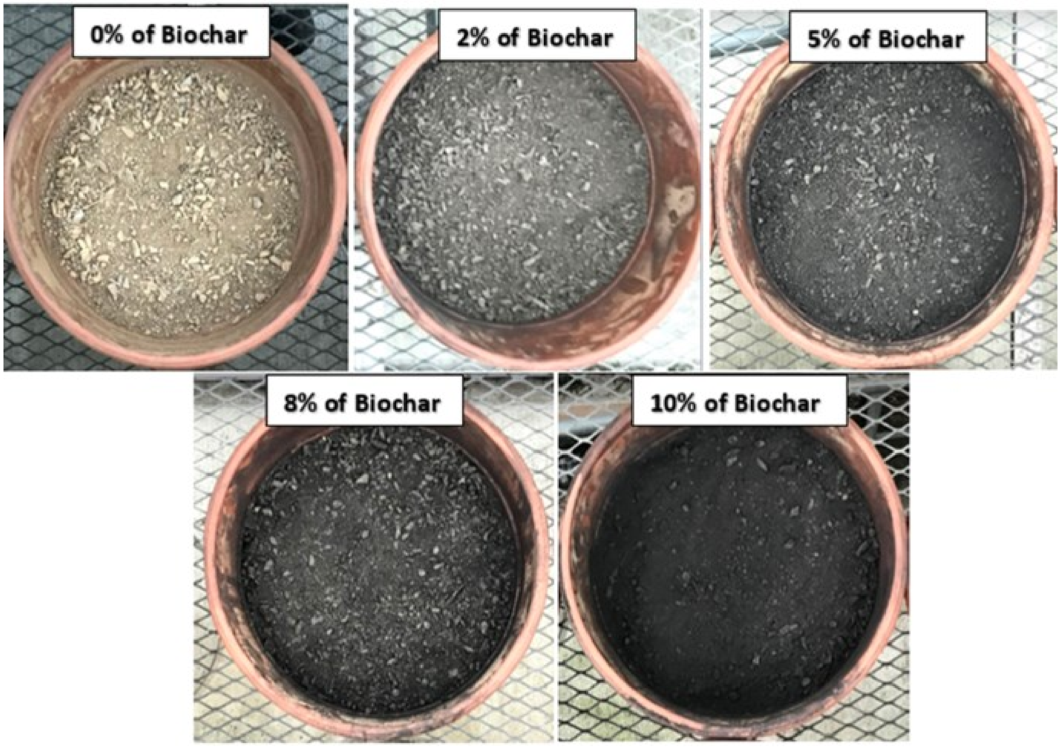

Figure S2. Soil-biochar mixtures prepared at five application rates, which were added as percentages based on the soil volume [v/v] (0, 2, 5, 8 and 10%) in the top 10 cm of the soil

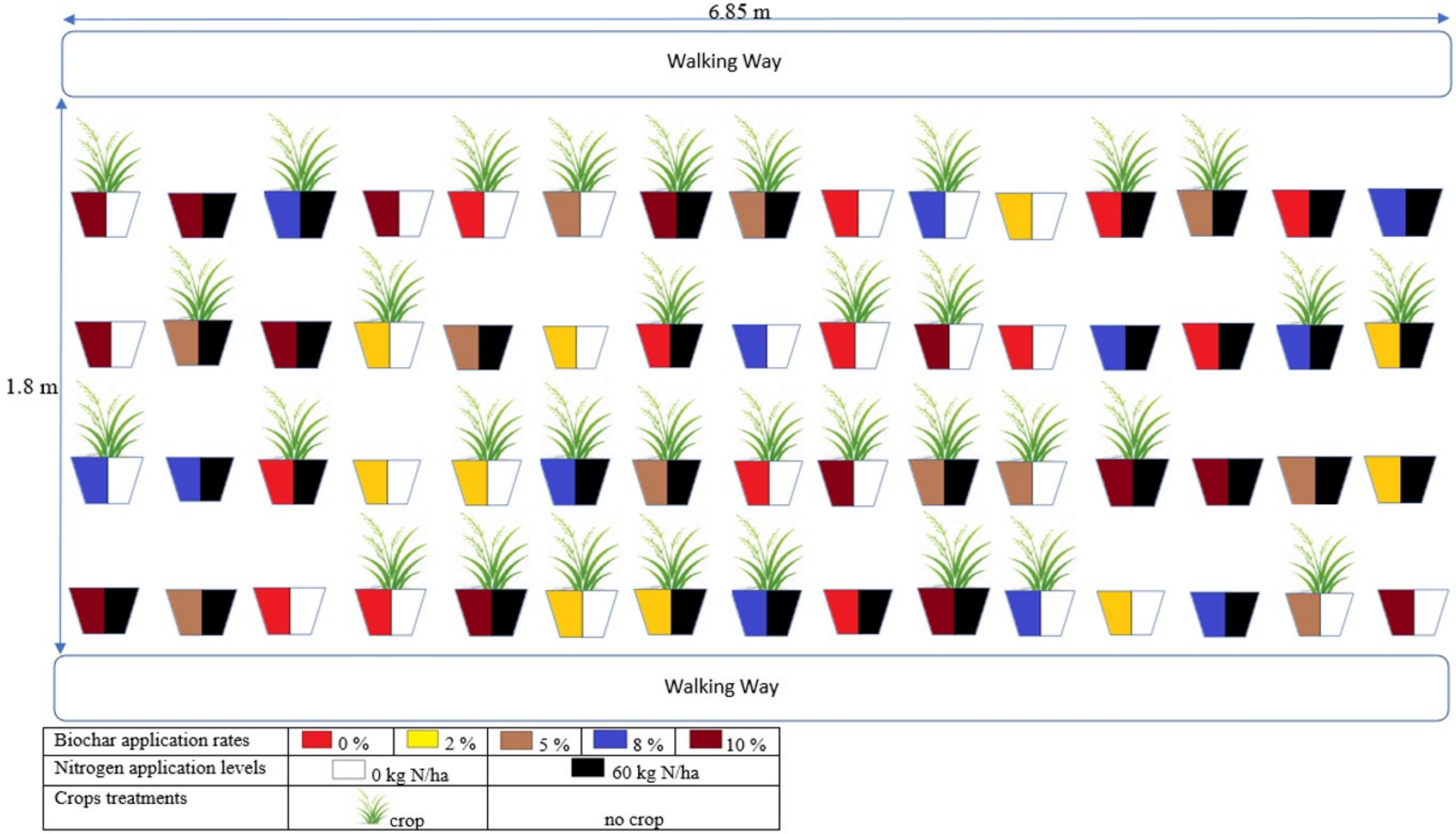

Treatments codes	***Treatment Description***	***Treatments codes***	***Treatment Description***	***Treatments codes***	***Treatment Description***	***Treatments codes***	***Treatment Description***
T_1	B_0%-N_0-crop	T_6	B_0%-N_{60}-crop	T_{11}	B_0%-N_0-no crop	T_{16}	B_0%-N_{60}-no crop
T_2	B_2%-N_0-crop	T_7	B_2%-N_{60}-crop	T_{12}	B_2%-N_0-no crop	T_{17}	B_2%-N_{60}-no crop
T_3	B_5%-N_0-crop	T_8	B_5%-N_{60}-crop	T_{13}	B_5%-N_0-no crop	T_{18}	B_5%-N_{60}-no crop
T_4	B_8%-N_0-crop	T_9	B_8%-N_{60}-crop	T_{14}	B_8%-N_0-no crop	T_{19}	B_8%-N_{60}-no crop
T_5	B_{10}%-N_0-crop	T_{10}	B_{10}%-N_{60}-crop	T_{15}	B_{10}%-N_0-no crop	T_{20}	B_{10}%-N_{60}-no crop

Figure S3. Experimental layout, showing biochar, nitrogen, and crop levels and the treatment descriptions within a completely randomized design (CRD)

Three-way ANOVA analysis equation.

$$Y_{ijk} = \mu + A_i + B_j + C_k + (AB)_{ij} + (AC)_{ik} + (BC)_{jk} + (ABC)_{ijk} + \varepsilon_{ijkl} \quad \text{(S1)}$$

Where Y_{ijk} is the dependent variable, μ is the overall mean, A_i, B_j, *and* C_k are the effects of *ith* (biochar rates), *jth* (nitrogen levels), and *kth* (crop treatments), respectively, and ε_{ijkl} is the random error term within the experiment.

Table S1. Analysis of variance (ANOVA) of estimated soil leachate pH in response to the main effects of biochar, nitrogen levels, crop, and interactions

Source of Variance	*df*	*Adj SS*	*Adj MS*	*F-Value*	*P-Value*
B % (v/v)	4	0.57612	0.144030	25.37	0.000
N (Kg/ha)	1	0.01896	0.018963	3.34	0.075
Crop	1	0.00600	0.006000	1.06	0.310
B % (v/v)*N (Kg/ha)	4	0.08527	0.021319	3.75	0.011
B % (v/v)*Crops	4	0.10307	0.025767	4.54	0.004
N (Kg/ha)*Crops	1	0.30057	0.300570	52.93	0.000
B % (v/v)*N (Kg/ha)*Crops	4	0.16615	0.041536	7.32	0.000
Error	40	0.22713	0.005678		
Total	59	1.48327			

Statistically significant difference ($P < 0.05$). Source of variance codes, including biochar (B), nitrogen (N), and crop.

Table S2. Analysis of variance (ANOVA) of estimated soil leachate EC (µs/cm) in response to the main effects of biochar, nitrogen levels, crop, and interactions

Source of Variance	*df*	*Adj SS*	*Adj MS*	*F-Value*	*P-Value*
B % (v/v)	4	0.11142	0.027856	6.53	0.000
N (Kg/ha)	1	0.37985	0.037985	89.09	0.000
Crop	1	0.33690	0.336900	79.01	0.000
B % (v/v)*N (Kg/ha)	4	0.00601	0.001504	0.35	0.841
B % (v/v)*Crops	4	0.00897	0.002243	0.53	0.717
N (Kg/ha)*Crops	1	0.09728	0.097284	22.82	0.000
B % (v/v)*N (Kg/ha)*Crops	4	0.04048	0.010120	2.37	0.068
Error	40	0.17056	0.004264		
Total	59	1.15148			

Statistically significant difference ($P < 0.05$). Source of variance codes, including biochar (B), nitrogen (N), and crop.

Table S3. Analysis of variance (ANOVA) of estimated soil leachate nitrate (mg/L) in response to the main effects of biochar, nitrogen levels, crop, and interactions

Source of Variance	*df*	*Adj SS*	*Adj MS*	*F-Value*	*P-Value*
B % (v/v)	4	654.89	163.72	102.60	0.000
N (Kg/ha)	1	3630.82	3630.82	2275.43	0.000
Crop	1	2373.68	2373.68	1487.58	0.000
B % (v/v)*N (Kg/ha)	4	37.49	9.37	5.87	0.001
B % (v/v)*Crop	4	3.83	0.96	0.60	0.665
N (Kg/ha)*Crop	1	0.18	0.18	0.11	0.740
B % (v/v)*N (Kg/ha)*Crop	4	24.20	6.05	3.79	0.010
Error	40	63.83	1.60		
Total	59	6788.90			

Statistically significant difference ($P < 0.05$). Source of variance codes, including biochar (B), nitrogen (N), and crop.

Table S4. Analysis of variance (ANOVA) of estimated soil leachate ammonium (mg/L) in response to the main effects of biochar, nitrogen levels, crop, and interactions

Source of Variance	*df*	*Adj SS*	*Adj MS*	*F-Value*	*P-Value*
B % (v/v)	4	69.899	17.475	55.51	0.000
N (Kg/ha)	1	194.965	194.965	619.31	0.000
Crop	1	0.386	0.386	1.23	0.275
B % (v/v)*N (Kg/ha)	4	49.712	12.428	39.48	0.000
B % (v/v)*Crop	4	3.002	0.751	2.38	0.067
N (Kg/ha)*Crop	1	0.132	0.132	0.42	0.521
B % (v/v)*N (Kg/ha)*Crop	4	1.629	0.407	1.29	0.289
Error	40	12.592	0.315		
Total	59	332.318			

Statistically significant difference ($P < 0.05$). Source of variance codes, including biochar (B), nitrogen (N), and crop.

Table S5. Analysis of variance (ANOVA) of estimated soil leachate total nitrogen (mg/L) in response to the main effects of biochar, nitrogen levels, crop, and interactions

Source of Variance	*df*	*Adj SS*	*Adj MS*	*F-Value*	*P-Value*
B % (v/v)	4	6141.1	1535.3	140.19	0.000
N (Kg/ha)	1	30260.1	30260.1	2763.20	0.000
Crop	1	8512.3	8512.3	777.30	0.000
B % (v/v)*N (Kg/ha)	4	2335.3	583.8	53.31	0.000
B % (v/v)*Crops	4	376.6	94.2	8.60	0.000
N (Kg/ha)*Crops	1	2139.5	2139.5	195.37	0.000
B % (v/v)*N (Kg/ha)*Crops	4	82.1	20.5	1.87	0.134
Error	40	438.0	11.0		
Total	59	50285.1			

Statistically significant difference ($P < 0.05$). Source of variance codes, including biochar (B), nitrogen (N), and crop

Table S6. Analysis of variance (ANOVA) of estimated soil leachate dissolved organic carbon (mg/L) in response to the main effects of biochar, nitrogen levels, crop, and interactions

Source of Variance	*df*	*Adj SS*	*Adj MS*	*F-Value*	*P-Value*
B % (v/v)	4	15599.8	3900.0	8.86	0.000
N (Kg/ha)	1	18596.7	18596.7	42.23	0.000
Crop	1	12000.1	12000.1	27.25	0.000
B % (v/v)*N (Kg/ha)	4	283.9	71.0	0.16	0.957
B % (v/v)*Crops	4	1705.6	426.4	0.97	0.435
N (Kg/ha)*Crops	1	4092.2	4092.2	9.29	0.004
B % (v/v)*N (Kg/ha)*Crops	4	69.7	17.4	0.04	0.997
Error	40	17613.0	440.3		
Total	59	69961.1			

Statistically significant difference ($P < 0.05$). Source of variance codes, including biochar (B), nitrogen (N), and crop

Table S7. Correlation coefficients between the leached parameters, including nitrate, ammonium, total nitrogen, total dissolved carbon, pH, and EC (n = 60)

	Nitrate (mg/L)	Ammonium (mg/L)	Dissolved organic carbon (mg/L)	Total nitrogen (mg/L)	pH
Ammonium (mg/L)	0.691<br>0.000***				
Dissolved organic carbon (mg/L)	0.358<br>0.005**	0.668<br>0.000***			
Total nitrogen (mg/L)	0.964<br>0.000***	0.786<br>0.000***	0.467<br>0.000***		
pH	-0.296<br>0.022*	-0.479<br>0.000***	-0.573<br>0.000***	-0.342<br>0.007**	
EC	0.853<br>0.000***	0.573<br>0.000***	0.324<br>0.012*	0.817<br>0.000***	0.198<br>0.130

*Correlation is significant (p-value < 0.05), **correlation is significant (p-value < 0.01), and ***correlation is significant ($p < 0.001$)

Table S8. Mean values of Final soil pH, % N, % C, % SOM, and CEC in response to biochar, nitrogen fertilizer and crop treatments used in the experiment.

Treatments	Soil pH	% N	% C	% SOM	CEC (cmol/Kg
B0%-N0-C	6.1 d	0.5	6.70 r	6.19 m	17.4 ef
B2%-N0-C	6.3 cd	0.6	11.0 n	8.58 a	17.9 cd
B5%-N0-C	6.3 cd	0.6	16.9 i	6.96 j	17.1 fg
B8%-N0-C	6.4 bcd	0.6	16.0 j	8.47 b	17.4 ef
B10%-N0-C	6.4 bcd	0.6	18.5 f	6.97 j	18.3 ab
B0%-N60-C	6.1 d	0.5	7.10 q	6.62 l	17.6 de
B2%-N60-C	6.2 d	0.6	10.7 o	7.49 g	16.5 h
B5%-N60-C	6.3 cd	0.6	17.1 h	7.95 e	14.6 k
B8%-N60-C	6.3 cd	0.6	13.8 k	6.87 k	16.0 i
B10%-N60-C	6.3 cd	0.6	19.0 e	8.03 d	17.7 cde
B0%-N0-NC	6.3 cd	0.6	7.80 p	7.66 f	17.1 fg
B2%-N0-NC	6.6 abc	0.6	11.8 l	8.47 b	15.6 j
B5%-N0-NC	6.7 ab	0.6	18.2 g	7.36 h	18.0 bc
B8%-N0-NC	6.9 a	0.7	19.7 d	7.23 i	17.5 e
B10%-N0-NC	6.8 a	0.6	21.3 a	6.97 j	17.4 ef
B0%-N60-NC	6.3 d	0.6	7.80 p	7.63 f	17.9 cd
B2%-N60-NC	6.6 abc	0.6	11.3 m	7.61 f	18.6 a
B5%-N60-NC	6.6 abc	0.6	18.4 f	8.36 c	17.0 g
B8%-N60-NC	6.8 a	0.6	20.0 c	6.95 j	16.4 h
B10%-N60-NC	6.8 a	0.6	20.7 b	6.67 l	17.6 de

Mean values followed by the same letter within a column are not significantly different ($p < 0.05$, Tukey's test). Treatments include five biochar applications (B0%, B2%, B5%, B8%, and B10%), nitrogen levels are (N0 and N60), and crop levels are: crop (C) and no crop (NC).
